# Behavioral components define operational suitability metric for detection dog success

**DOI:** 10.3389/fvets.2026.1887925

**Published:** 2026-07-27

**Authors:** Jingyi Zheng, Lucia Lazarowski, Abbigail L. Lanier, Pamela S. Haney, Emily M. Chester, Paul Waggoner, Xu Wang

**Affiliations:** 1 Department of Mathematics and Statistics, College of Science and Mathematics, Auburn University, Auburn, AL, United States; 2 Canine Performance Sciences Program, College of Veterinary Medicine, Auburn University, Auburn, AL, United States; 3 Department of Anatomy, Physiology and Pharmacology, College of Veterinary Medicine, Auburn University, Auburn, AL, United States; 4 Department of Psychological Sciences, College of Liberal Arts, Auburn University, Auburn, AL, United States; 5 Department of Pathobiology, College of Veterinary Medicine, Auburn University, Auburn, AL, United States; 6 Detection Canine Science, Innovation, Technology and Education Program, College of Veterinary Medicine, Auburn University, Auburn, AL, United States; 7 Center for Advanced Science, Innovation and Commerce, Alabama Agricultural Experiment Station, Auburn, AL, United States; 8 HudsonAlpha Institute for Biotechnology, Huntsville, AL, United States; 9 Scott-Ritchey Research Center, College of Veterinary Medicine, Auburn University, Auburn, AL, United States

**Keywords:** Auburn University dog, behavioral component score, computationally synthesized suitability, co-occurrence network, detection performance, environmental soundness, random forest, working dog

## Abstract

**Introduction:**

To ensure effective development and assessment of detection dogs, Auburn University Canine Performance Sciences program has implemented a structured behavioral evaluation at four developmental milestones.

**Methods:**

At each timepoint, trainers scored candidate detection dogs on 11 subtests evaluating detection performance and environmental soundness using behavioral component (BC) scores. Additionally, trainers rated each dog’s overall suitability for operational detection work using a singular global score. To investigate the relative importance of different metrics in predicting final outcomes, we leveraged a longitudinal dataset collected from 304 dogs.

**Results:**

Using a machine learning classifier, we discovered that suitability (S) scores at 12-months of age were highly predictive of training outcomes, achieving a prediction accuracy of 74.3%. The S scores appear to reflect a synthesis of subtest scores across relevant behavioral domains, illustrated by their correlative structures. To determine if S scores could be replicated using only BC scores, we estimated computationally synthesized suitability (CSS) scores at each timepoint using five significant BC scores as predictors in a linear model. Strong correlations were observed between trainer-reported suitability and CSS, with spearman’s correlation coefficients exceeding 0.84 at all timepoints, demonstrating CSS scores’ excellent ability to predict suitability. As predictors of training outcome, trainer-reported S scores demonstrated high accuracy, while CSS scores exhibited high precision (76.7%). Notably, both suitability metrics increase over time in dogs deemed operationally capable but not in unsuccessful dogs.

**Discussion:**

Our findings provide novel insights into the importance of data-driven approaches by combining expert trainer global assessments of suitability and BC scores with computational modeling in detection dog development.

## Introduction

Detection canines are dogs purposely trained to identify numerous potential threats, including narcotics (1), explosives (2), pathogens (3), and invasive species (4, 5). Deployment of successful detection dogs is crucial to national security, law enforcement, and the agricultural industry, and the demand for capable dogs continues to rise (6). However, the significant time and costs invested in their development and subsequent selection, coupled with substantial attrition from training, present significant challenges to the widespread application of successful detection dogs as well as enhancement of their capabilities.

Selection of detection dogs ideally suited for specific working roles relies primarily on assessments of behavioral characteristics (7, 8). Behavioral evaluations which observe dogs’ responses to various stimuli or environmental contexts are often used to characterize relevant traits (9–11). These evaluations typically include a set of standardized tests in which the dog is given an ordinal score indicating gradations of an observed characteristic (12, 13), or the duration and frequency of specific behaviors can be coded and used to inform selection (14). Many different characteristics have been linked to the successful working performance of detection dogs, including reward engagement (10), motivation to work (1), trainability (9) and lack of fear to novel or startling stimuli (12).

In detection dogs, suitability for operational work requires the presence of specific behavioral phenotypes across multiple domains. Namely, detection dogs must demonstrate characteristics associated with detection performance (e.g., task engagement, effective search techniques, cooperation with the handler) and environmental soundness (e.g., ability to navigate high stimulus environments or obstacles (15–17)). The appropriate combination of these specific traits ensures that the dog exhibits stable and consistent detection performance in dynamic operational environments. A single metric serving as an overall assessment of a dog’s suitability for operational detection work may provide a simple but effective metric of a dog’s ability to succeed in the training program, but accurate representations of this metric would require input from experienced canine trainers that understand the necessary phenotypes for operational suitability. In addition, at different points in development, certain characteristics may have greater weight in judgments of suitability, so understanding how these assessments may change over time is also important to measure. Past studies evaluating characteristics associated with suitability for detection work have discussed the relevance of separate characteristics in determining the success of detection dogs (1, 17–19); however, there has been little evaluation of how overall assessments of suitability for operational work by trainers or evaluators may predict outcome in a training program, and how these assessments may factor in separate behavioral domains at various points across development.

Auburn University College of Veterinary Medicine Canine Performance Sciences (AUCVM-CPS) have conducted standardized behavioral assessments consisting of a series of subtests that measure behaviors relevant to the operational performance of purpose-bred candidate detection dogs. In addition to trainers providing behavioral component (BC) scores on each subtest, which are designed to capture specific behavioral responses related to detection capabilities and environmental soundness, they also assign a singular global measure of dogs’ suitability (S) for operational detection work based on their overall impression of the dogs’ behavior throughout the evaluation. These two different approaches of assessing dogs’ capabilities allow for a comparison of the accuracy of each method to examine potential advantages and disadvantages of more discretionary, expertise-based assessment versus more prescribed, systematic measurement. It also provides an opportunity to assess how global assessments of operational suitability may synthesize separate components of the behavioral evaluation at different timepoints. Added to the advantage are the standardized rearing conditions and training programs at AUCVM-CPS colony (20), providing unique opportunities to investigate various research questions in a controlled setting.

In this study, we leveraged longitudinal phenotypic data from more than 300 detection dogs in the AUCVM-CPS colony to systematically investigate the relationships among the global S score, multiple BCs related to detection dog performance, and the final training outcomes. Specifically, we examined how BCs scored throughout development associate with the transition into operational elite detection work, and/or selection as an elite breeder, collectively defined as operationally capable (OC). Although these analyses were based on an early version of BCs and scoring methodology, they provided a unique opportunity to evaluate their predictive value and relative importance of longitudinal behavioral assessments in one of the world’s most comprehensively phenotyped detection canine populations. The findings provide a quantitative framework for understanding the behavior factors associated with detection dog training success and establish a foundation for enhancing the phenotype-based selection and training strategies.

## Materials and methods

### Subjects and ethics statement

Dogs enrolled in this study (*N* = 304 dogs in *N* = 48 litters) were raised at the Auburn University College of Veterinary Medicine Canine Performance Sciences (AUCVM-CPS) under the same diet, training, and medical care in a controlled environment (Data S1). Dogs were Labrador retrievers and Labrador retriever (LR) x German wirehaired pointer crosses (GWP) (7). All experimental animal protocols were approved by the Institutional Animal Care and Use Committee (IACUC) of Auburn University.

### Training outcomes

The training outcomes of dogs bred and trained at the AUCVM-CPS colony were determined based on their suitability for an operational detection role at the conclusion of training at approximately one to one and a half years of age (Data S1). All dogs purchased and successfully placed into operational service by clients (sale quality dogs, SQ) were labeled as “Operationally Capable” (OC). Dogs that were retained in the AUCVM-CPS colony for breeding purposes, which were selected based on their demonstrably superior potential detection performance, were also categorized as OC. Dogs that were not sold or were returned by clients due to performance deficiencies, were labeled as “Washout” (WO). A small proportion of the WO dogs were due to medical reasons (*N* = 24).

### Longitudinal behavioral evaluations

As part of the training program at AUCVM-CPS, each puppy was evaluated by 1–3 AUCVM-CPS trainers at four timepoints: 3-month, 6-month, 10-month, and 12-month (7). At each timepoint, a standardized behavioral assessment composed of various subtests was conducted in which trainers assigned dogs a series of 13 behavioral component (BC) scores. The 7 performance-related behavioral component phenotypes (P-BC) are Air Scenting, Work/Effort, Independence, Physical Possessiveness of Toy (Toy. P), Focus on Task/Reward (Task. F), Retrieve, and Hunt. The 6 environmental soundness-related behavioral component phenotypes (E-BC) are Surfaces, People, Vehicles/Urban Clutter, Visual Startle, Acoustic Startle, and Excitability. Additionally, at the completion of the evaluation, the trainer gave an assessment of the dog’s overall capability to perform operational detection work designated as the suitability (S) score, which was previously referred to as “Trainability” in Lazarowski et al., 2018. This score differed from the BC scores in that it was not tied to specific sub-tests of the evaluation, but rather, it was intended to reflect the trainer’s overall judgment of that dog’s capability for operational work based on its performance throughout the entire evaluation. All behavioral metrics were measured on a 5-point ordinal scale from 1 to 5, with 1 being the least and 5 being the most desirable expression of the metric (Supplementary Figure S1). Uncollected data was recorded as 0 (Data S2). Detailed definitions of these phenotypic assessments were published as AUCVM-CPS Detection Canine Behavioral Phenotype Evaluation System version 1.0 (DCBPES V1.0) (7). Starting from December 2018, AUCVM-CPS transitioned to Detection Canine Behavioral Phenotype Evaluation System version 2.0 (DCBPES V2.0) (21). Approximately 4% of the behavioral evaluation scores (Data S2) were collected using DCBPES V2.0 from 19 dogs born immediately before the 2018 transition from V1.0 to V2.0 (10- and 12-month timepoints only). Although there were some minor definitional and scoring differences between V1.0 and V2.0 (21), the majority of underlying behavioral constructs were consistent across versions (e.g., Air Scenting vs. Air Scenting, Focus on Toy/Reward vs. Reward Focus, People vs. People, and Vehicles & Urban Clutter vs. Traffic). The inclusion of these V2.0 scores likely only slightly eroded the observed correlational structures with minor effects.

### Correlation between suitability scores and BC scores

**Figure 1:**
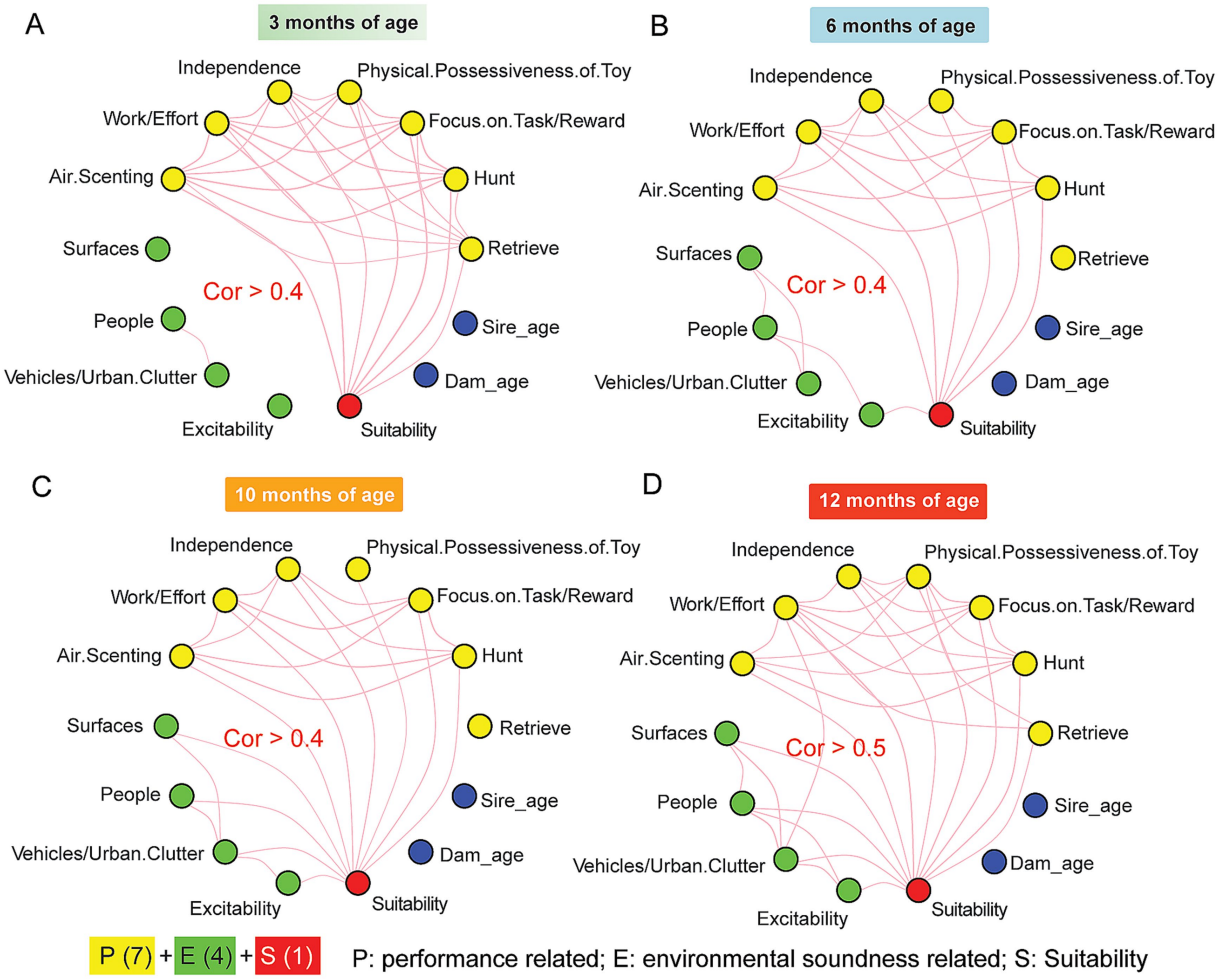
Correlative structures among suitability scores and 11 behavioral component (BC) scores at four key developmental timepoints. Correlated network plots demonstrating significant correlations among behavioral metrics at the 3- month **(A)**, 6-month **(B)**, 10-month **(C)**, and 12-month **(D)** timepoints. Performance-related (P-BC) metrics, including Air. Scenting, Work/Effort, Independence, Physical Possessiveness of Toy, Focus on Task/Reward, Retrieve, and Hunt, are represented in yellow circles. Environmental soundness-related (E-BC) metrics, including Surfaces, People, Vehicles/Urban Clutter, and Excitability, are represented in green circles. The red circle represents the suitability score. The paternal age and maternal age at birth are represented by blue circles. Statistically significant Spearman’s correlations exceeding the predefined threshold (0.4 or 0.5) are depicted by edges connecting the respective variables. The color of the edge indicates the sign of the correlation, with red representing positive correlations and blue indicating negative correlations.

**Figure 2:**
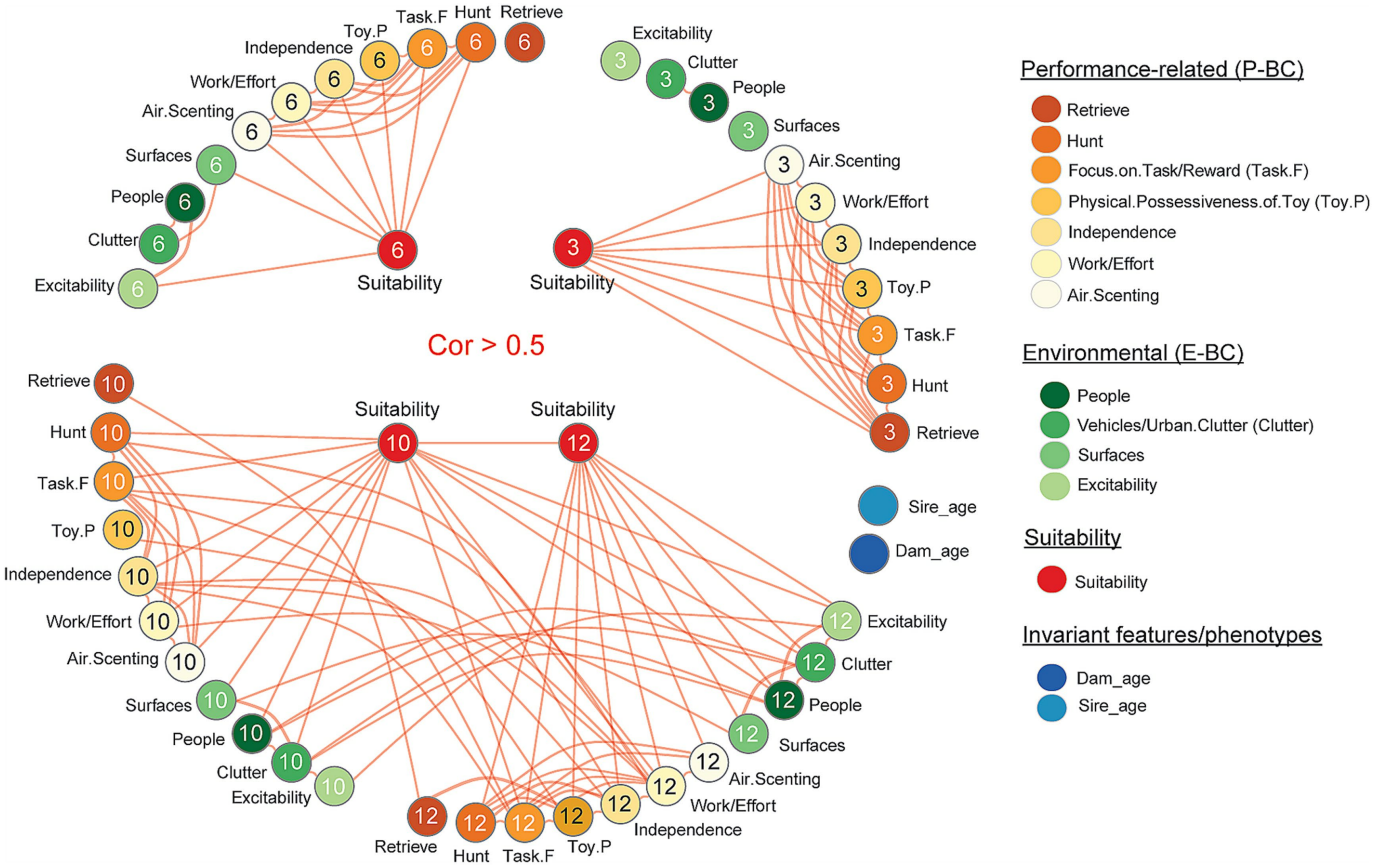
Inter-developmental timepoints correlative structures of 12 behavioral metrics. Correlation network plots demonstrating significant correlations among suitability scores and 11 behavioral component (BC) scores at the 3-month, 6-month, 10-month, and 12-month timepoints. Performance-related metrics (P-BC), including Air. Scenting, Work/Effort, Independence, Physical Possessiveness of Toy, Focus on Task/Reward, Retrieve, and Hunt, are represented by circles color-coded using a yellow-brown spectrum. Environmental soundness-related metrics (E-BC), including Surfaces, People, Vehicles/Urban Clutter, and Excitability, are represented in green circles. Red circles represent suitability scores, which are moved inside the circle for better visualization. The paternal age and maternal age at birth are represented by blue circles. Statistically significant Spearman’s correlations exceeding a predefined threshold of 0.5 are depicted by edges connecting the respective variables. The color of the edge indicates the sign of the correlation, with red representing positive correlations and blue indicating negative correlations.

We included the P-BC scores and four of the six E-BC scores to investigate the relationships between suitability (S) scores and other behavioral metrics. Two of the E-BC scores (Visual Startle and Acoustic Startle) were intentionally omitted for some timepoints (29 and 40% uncollected data respectively), and they were excluded from subsequent analysis. The remaining 12 metrics have 8.9% missing data on average (Data S2). Two invariant factors, dam’s age and sire’s age at birth, were also included in the analysis. We conducted correlation analyses with these 14 variables, and Spearman’s correlation coefficients were computed for each pair of the 14 variables. Statistical significance was assessed using permutation tests. To explore temporal fluctuations in the correlation structure, we conducted correlation analyses at the 3-month, 6-month, 10-month, and 12-month timepoints, respectively. Spearman’s correlation networks were constructed to visualize the relationships among suitability and BC phenotypes. To highlight moderate-to-strong associations while maintaining interpretable network structures, different correlation thresholds were applied depending on the analysis. A threshold of |*ρ*| > 0.4 was used for the longitudinal networks spanning the 3-, 6-, 10-month timepoints to capture developmental connections, because at these stages the correlations were generally weaker (Figure 1). For the 12-month network, a more stringent threshold of |ρ| > 0.5 was applied to reduce network complexity and emphasize the strongest associations (Figure 1). Correlative networks were also examined for all timepoints combined (Figure 2).

### Random forest classification analysis

To assess the predictive capability of specific variables across timepoints, including all behavioral metrics and time-invariant phenotypes such as litter identity, color, sex, delivery type, evaluator, dam, and sire age, multiple random forest models (22) were developed. Random forest models were selected because they can capture nonlinear relationships, higher-order interactions among behavioral phenotypes, and correlated predictors without requiring strong distributional assumptions. These characteristics are particularly well suited for behavioral datasets, in which multiple interrelated phenotypes may jointly contribute to training outcomes. In addition, random forests provide robust measures of predictor importance, allowing the relative contributions of behavioral and non-behavioral variables to detection dog training success to be quantified.

In each model, the response variable was the binary training outcome (OC vs. WO). Predictor variables included the 11 BC scores, evaluator-reported suitability score, evaluator identity, and the time-invariant phenotypes. Recognizing potential shifts in the relative importance of predictors over time, four random forest models were built at the 3-month, 6-month, 10-month, and 12-month timepoints using corresponding metrics. At each timepoint, evaluation scores were determined by 1–3 evaluators (Data S2), and data entries with missing score(s) were excluded from the analysis. The sample size of remaining data points (sets of 11 BC scores with a suitability score) ranged from 323 to 490 complete evaluations across 304 dogs (Supplementary Figure S2). For every model, predictor importance scores were computed, ranking the predictors from most to least important. All models were evaluated using repeated cross validation with 20% data serving as testing data. In the case of random forest models, feature selection was guided by the importance of features measured by the mean decrease in the Gini index, and only the top-ranking features were chosen. Model performance metrics, including prediction accuracy, precision, sensitivity, and specificity, were computed in R software caret package. Accuracy measures the proportion of correctly classified dogs, precision measures the proportion of predicted OC dogs that were truly OC, sensitivity represents the proportion of OC dogs correctly identified, and specificity represents the proportion of WO dogs correctly identified.

Because these metrics evaluate complementary aspects of model performance, all four were reported to provide a comprehensive assessment of each predictive model.

**Figure 3:**
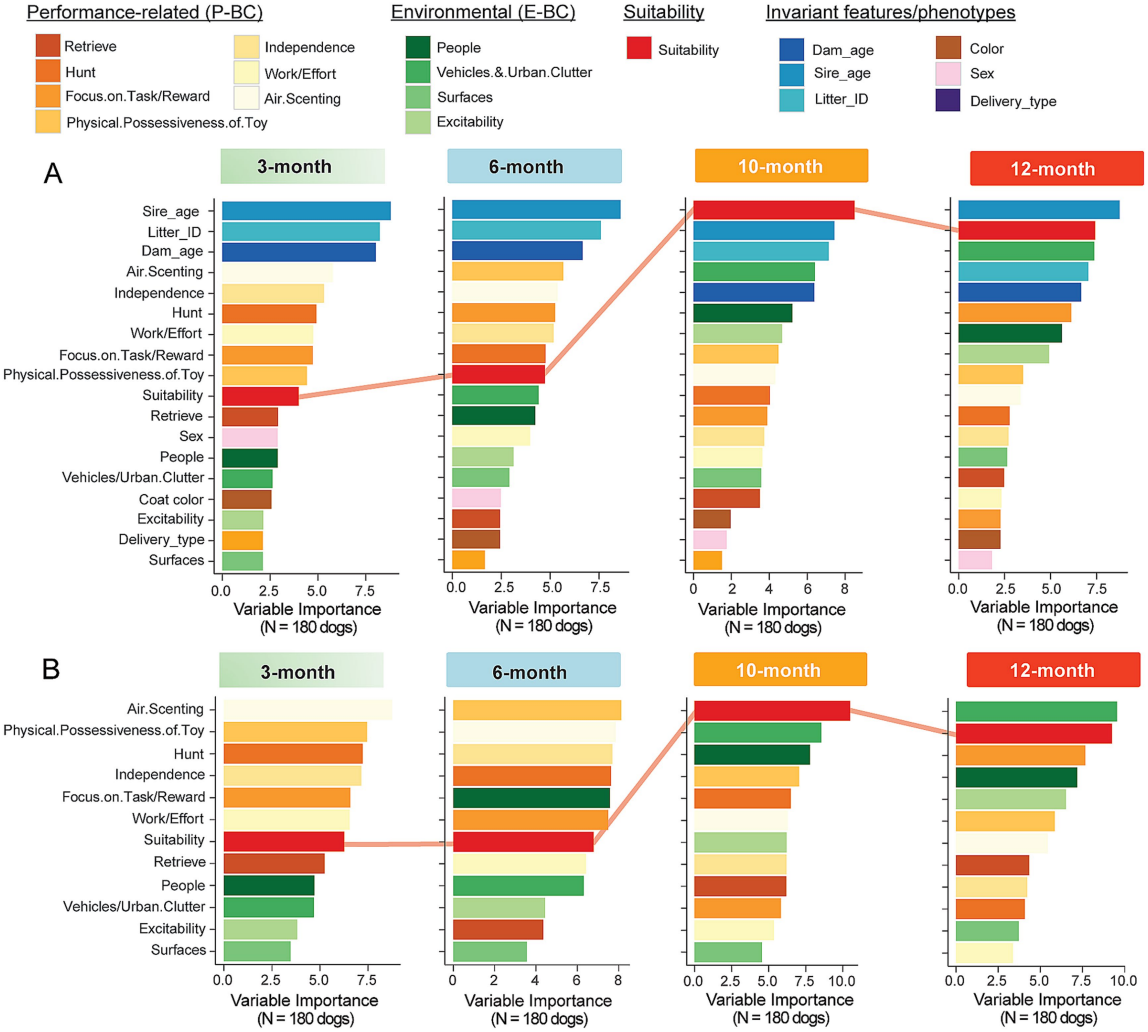
Longitudinal changes of feature importance of behavioral phenotypes in predicting training outcomes. **(A,B)** Barplots of feature importance scores computed using random forest classifier at the 3-month, 6-month, 10-month, and 12-month timepoints using all variables **(A)** and behavioral component (BC) phenotypes only **(B)** across all four timepoints. Longitudinal phenotypic measurements included in the model are 7 performance-related phenotypes based on behavioral component scores (P-BC), 4 environmental soundness-related phenotypes based on behavioral component scores (E-BC), and suitability scores. Invariant phenotypes and features included in panel **(A)** are dam and sire’s age at birth, litter ID, coat color, sex, delivery type, and evaluator. Shaded lines connect suitability scores (*red*) between timepoints to demonstrate its change in feature importance ranking during development.

Because no significant evaluator bias was identified (Supplementary Figure S2), assessments from multiple evaluators on the same dog were averaged to compute one evaluation score for each timepoint. Dogs with missing data at any of the four timepoints were excluded from the analysis, leaving a final training set consisting of 180 dogs. Based on this data set with complete measurements, random forest models were then refitted using all variables including the 11 averaged BC scores, averaged suitability scores, and time-invariant phenotypes such as litter identity, color, sex, and delivery type to predict training outcomes, and the relative feature importance was re-evaluated (Figure 3A). With only suitability scores and the 11 BC scores as input, the random forest models were retrained to predict training outcomes, and the relative feature importance was re-evaluated (Figure 3B).

### Computationally synthesized suitability modeling

Given the strong correlations between evaluator-reported suitability scores and the BC scores, separate linear regression models were developed at the 3-month, 6-month, 10-month, and 12-month timepoints to predict suitability scores from the 11 BC scores. Linear regression model was selected because its coefficients provide a simple and interpretable measure of the relative contribution of each BC to evaluator-reported suitability. Statistically significant BC predictors were retained in the final models to compute the predicted suitability score, which is defined as CSS (Computationally Synthesized Suitability), ranging from 0 to 5. Standard residual diagnostic plots were examined to assess the assumptions of linear regression, and no substantial violations were observed. In these models, evaluator-reported suitability served as the dependent variable, whereas the 11 BC scores were the independent variables. Time-invariant characteristics were not included in the regression models but were evaluated separately as candidate predictors in the random forest classification models rather than as adjustment covariates.

### Statistical analysis on longitudinal changes of suitability scores

To understand changes in suitability scores across timepoints, we conducted statistical analyses on the 180 dogs possessing complete sets of evaluation scores. Specifically, we examined whether longitudinal changes in suitability scores may differ between operationally capable (OC) and washout (WO) dogs. To quantify changes in suitability scores over time, we fitted separate linear regression models to the evaluator-reported suitability scores and the CSS scores of each dog across timepoints and recorded the slope. A positive slope indicates an increase in suitability scores across timepoints, while a zero or negative slope indicates no improvement or a decrease, respectively. Subsequently, we utilized the Mann–Whitney U test to assess whether the changes in suitability scores differed between operationally capable dogs and washouts.

## Results

### Cohort characteristics and longitudinal behavioral score distributions

Among the 304 dogs, 190 (62.5%) were deemed operationally capable (OC) dogs, and 114 (37.5%) were washouts (WO; Supplementary Figure S1A). No significant differences in OC rate were detected between the two sexes (Supplementary Figure S1B; *p* = 0.61, Fisher’s Exact Test), or the delivery type (C-section vs. free whelping, Supplementary Figure S1C; *p* = 0.73, Fisher’s Exact Test). In terms of coat color, 8.6% of the dogs were chocolate (*N* = 26), and 92.3% of them were OC (Supplementary Figure S1D; *p* = 0.0004, Fisher’s Exact Test). However, the significance arose from the presence of a small number of closely related chocolate dogs that happened to be OC. Excluding the chocolate dogs, the observed significance disappeared, and there was no notable difference in OC rate between black and yellow dogs (*p* = 0.59, Fisher’s Exact Test). The total number of behavioral evaluation scores for the 304 dogs was 24,133, spanning 11 BC categories and the one overall suitability measure at four timepoints (3-month, 6-month, 10-month, and 12-month). The distributions of all scores were centered around 3 (Supplementary Figure S1E).

### Correlation network analyses reveal the relationships between suitability scores and behavioral component phenotypes

The seven P-BCs consistently exhibited significant positive inter-correlations across all timepoints, and they were all connected to suitability (except Retrieve at the 6-month and 10-month; Figure 1). At the 3-month timepoint, E-BCs lacked inter-correlation, and they were not connected to suitability (Figure 1A). At the 6-month timepoint, E-BCs became inter-connected, but they were not connected to suitability yet, except for Excitability (Figure 1B). At later timepoints (10-month and 12-month), suitability demonstrated significant correlations to both P-BCs and E-BCs, and yet P-BCs and E-BCs remained two separate groups (Figures 1C,D), with the only exception being an edge between Work/Effort (P-BC) and Vehicles/Urban Clutter (E-BC) at the 12-month timepoint. Time-invariant features, dam age and sire age, were included as negative controls, as expected, showed no significant correlations with any BCs (Figure 1; correlation coefficient < 0.4). Therefore, suitability was a hub to connect the P-BC and E-BC modules defined based on intercorrelation, serving as an overall assessment of behavioral characteristics across both domains.

To assess correlations between suitability and BC scores at the four developmental timepoints, we examined the correlative structure of all behavioral measures at all timepoints in a single network (Figure 2). At the 3-month and 6-month timepoints, suitability and BC scores formed distinct stage-specific clusters with no crosstalk to later developmental timepoints (Figure 2). At the 10-month and 12-month timepoints, suitability variables occupied the center of their own clusters with the greatest number of connected edges, and they were significantly correlated to each other as well (Figure 2). In addition, strong interconnectivity between the 10-month and 12-month developmental timepoints was observed, revealing significant correlations across various behavioral components. First, 10-month suitability is connected to both 12-month P-BC (Work/Effort, Independence, and Focus on Task/Reward) and E-BCs (People, Vehicles/Urban Clutter, and Excitability). Second, there was a strong link between the 10- and 12-month P-BCs, mediated by 6 of the 7 P-BCs except for Air. Scenting (Figure 2). Independence at the 10-month timepoint was not only connected to 12-month Independence, but also 12-month Focus on Task/Reward and Work/Effort (Figure 2). Such multiple connectivity is also observed for 12-month Work/Effort, which is connected to 10-month Work/Effort, Independence, and Focus on Task/Reward (Figure 2). Finally, all 4 E-BCs were connected at the 10 and 12-month timepoints, suggesting a developmental continuity in environmental responses and stress tolerance at these two later stages (Figure 2).

### Behavioral component phenotypes, suitability and litter identity predict training outcomes, whereas coat color, sex, delivery type, and evaluator have minimal predictive importance

Random forest models (see Materials and Methods) revealed low feature importance for “Evaluator” at the 3-month and 6-month timepoints, and “Evaluator” had the least importance at the 10-month and 12-month timepoints (Supplementary Figure S2), suggesting that evaluator variation does not significantly influence the predictability of training outcome. Accordingly, potential evaluator effects were mitigated by averaging the scores, and subsequent analyses included 180 dogs without any missing data across all four timepoints (Data S3).

When all features were used, we achieved 65.89, 66.51, 70.62, and 74.65% prediction accuracy for the 3-month, 6-month, 10-month, and 12-month timepoints, respectively (Table 1). Regarding the relative feature importance at the 3-month and 6-month timepoints, the most influential factors are litter ID, dam age, and sire age (Figure 3A). These three factors consistently ranked the top three highest in feature importance, and they remained within the top five at the 10-month and 12-month timepoints in predicting the training outcomes (Figure 3A). Parental age and litter ID are inherently linked in this dataset, as puppies from the same litter share both attributes. Therefore, the effect of parental age is likely to be mediated by litter identity rather than representing an independent factor. Other time-invariant features, such as sex, coat color, and delivery type, displayed minimal predictive value in these models (Figure 3A).

**Table 1:** Training outcome prediction accuracy and precision using different sets of phenotypic data at 3-month, 6-month, 10-month, and 12-month timepoints.

Model performance metrics	Predictors	3-month	6-month	10-month	12-month
Accuracy	All (Random Forest)	65.89%	66.51%	70.62%	74.65%
	P-BC + E-BC + S	59.18%	62.81%	69.13%	76.56%
	Performance (P-BC)	57.65%	58.69%	60.71%	68.40%
	Environmental (E-BC)	59.24%	63.53%	61.81%	69.42%
	Suitability (S)	63.22%	65.31%	70.47%	74.31%
	CSS (Linear)	52.78%	58.89%	58.33%	61.67%
Precision	All (Random Forest)	71.15%	72.60%	73.74%	79.46%
	P-BC + E-BC + S	64.37%	67.04%	71.21%	76.25%
	Performance (P-BC)	64.23%	64.26%	66.10%	70.59%
	Environmental (E-BC)	63.44%	67.03%	67.14%	71.43%
	Suitability (S)	64.62%	65.14%	72.27%	74.23%
	CSS (Linear)	65.31%	70.71%	71.28%	76.74%

CSS: predicted suitability scores based on a combination of 5 selected performance-related (P-BC) and environmental soundness-related behavioral component phenotypes (E-BC).

### Evaluator-reported suitability score is the strongest predictor for training outcomes

Using only behavioral assessments (suitability and BC scores), training outcome prediction accuracy increased with age from 59.2% at 3-month to 76.6% at 12-month timepoint (Table 1). At 12 months, suitability alone predicted training outcomes (74.3%) nearly as accurately as all features combined (74.7%), and more accurately than P-BCs (68.4%) or E-BCs (69.4%). In the random forest models, we discovered that suitability had moderate feature importance at the 3-month and 6-month timepoints, and it ranked the highest in feature importance at the 10-month timepoint and remained within the top two most important predictors at the 12-month timepoint (Figure 3B). These findings demonstrate that evaluator-reported suitability is a robust predictor of detection dog training outcomes, particularly during the later stages of development.

### Computationally synthesized suitability accurately recapitulates suitability and its developmental relationships with behavioral components and training outcomes

**Figure 4:**
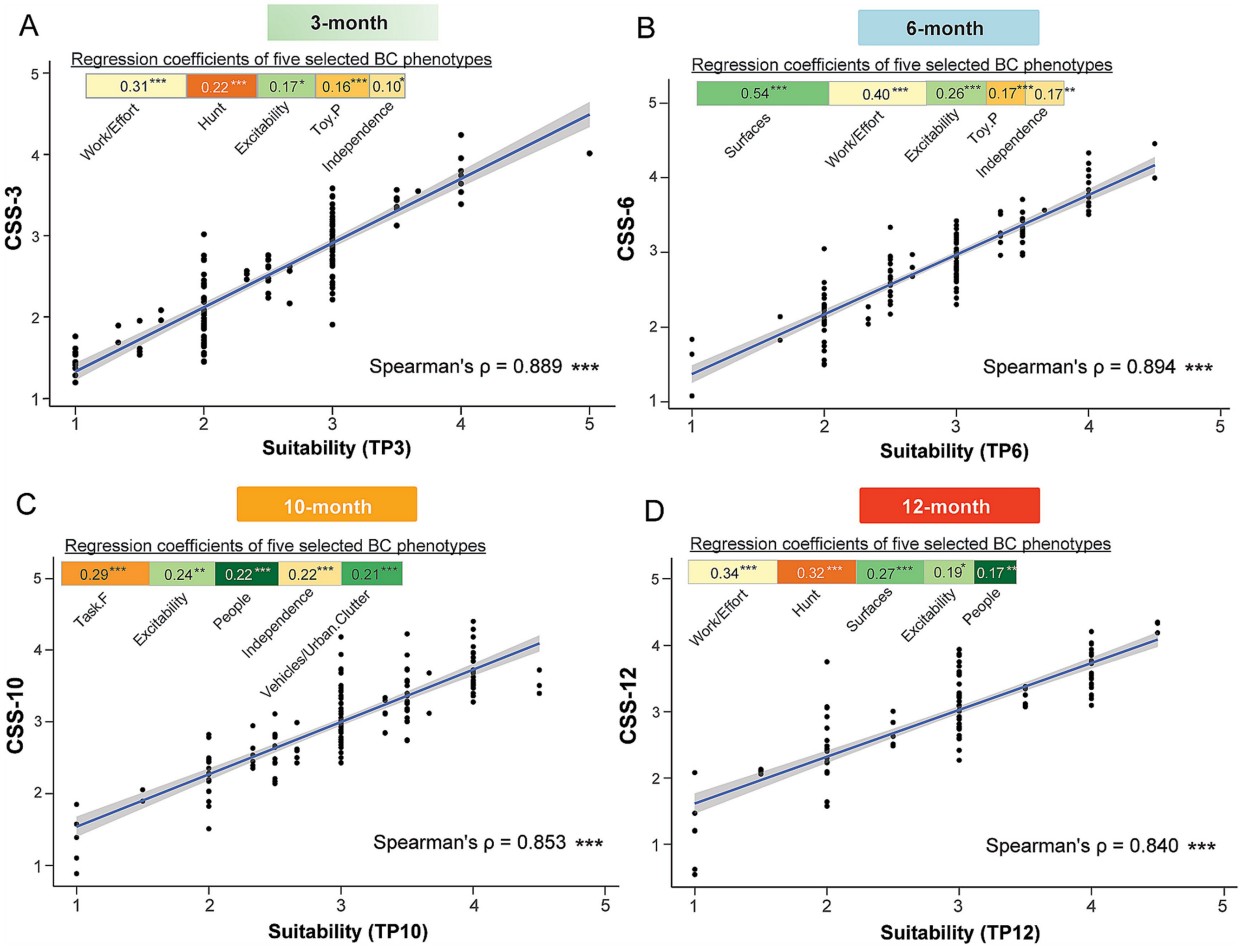
Computationally synthesized suitability (CSS) scores and their performance in predicting training outcomes. Scatterplots of computationally synthesized suitability (CSS) scores on the *y*-axis against the evaluator-reported suitability on the *x*-axis at the 3-month **(A)**, 6-month **(B)**, 10-month **(C)**, and 12-month **(D)** timepoints. CSS-3, CSS-6, CSS-10, and CSS-12 are predicted using linear regression models. A fitted regression line is plotted to demonstrate the trend and a non-parametric Spearman’s correlation coefficient is computed between CSS and suitability scores. A horizontal barplot of regression coefficients was drawn to show the relative weight of five selected behavioral component phenotypes utilized to estimate CSS scores. Statistical significance: *p < 0.05; **p < 0.01; ***p < 0.001.

Since suitability was highly correlated with BCs (Figure 1), we developed linear regression models that identified five significant BCs for predicting suitability scores across four timepoints (Figure 4; Supplementary Table S1). The predicted suitability scores, defined as Computationally Synthesized Suitability (CSS; CSS-3, CSS-6, CSS-10, and CSS-12), showed strong agreement with suitability scores across all four developmental timepoints (Spearman’s *ρ* = 0.840–0.894; Figure 4). Although five statistically significant BC predictors varied across timepoints (Figure 4), several consistent patterns emerged. Each model included both P-BCs and E-BCs, with P-BCs contributing more than E-BCs at early stages (82% vs. 18% relative weight at the 3-month timepoints) but becoming equally important at the 12-month timepoint (51% vs. 49%; Figure 4). Excitability was a significant predictor at all four timepoints (*p* < 0.05; Supplementary Table S1), whereas Work/Effort and Independence were significant at three of the four timepoints (*p* < 0.05; Supplementary Table S1).

To evaluate whether CSS preserved the relationship between suitability and BCs, we performed network analysis for all BCs by replacing evaluator-reported suitability with CSS (Supplementary Figures S3, S4). CSS maintained correlation patterns highly similar to suitability score, with 19 of the 20 predictor BCs showing significant correlations in both suitability (Figure 2) and CSS networks (Supplementary Figure S3). Moreover, CSSs retained comparable connectivity with BCs that were not used in the models (Supplementary Figure S3), indicating that it faithfully recapitulates the behavioral correlation structure of suitability, presumably through partial correlations among the BCs. The only exception was CSS-12, which had several additional connections to 10-month BCs (Supplementary Figure S3).

**Figure 5:**
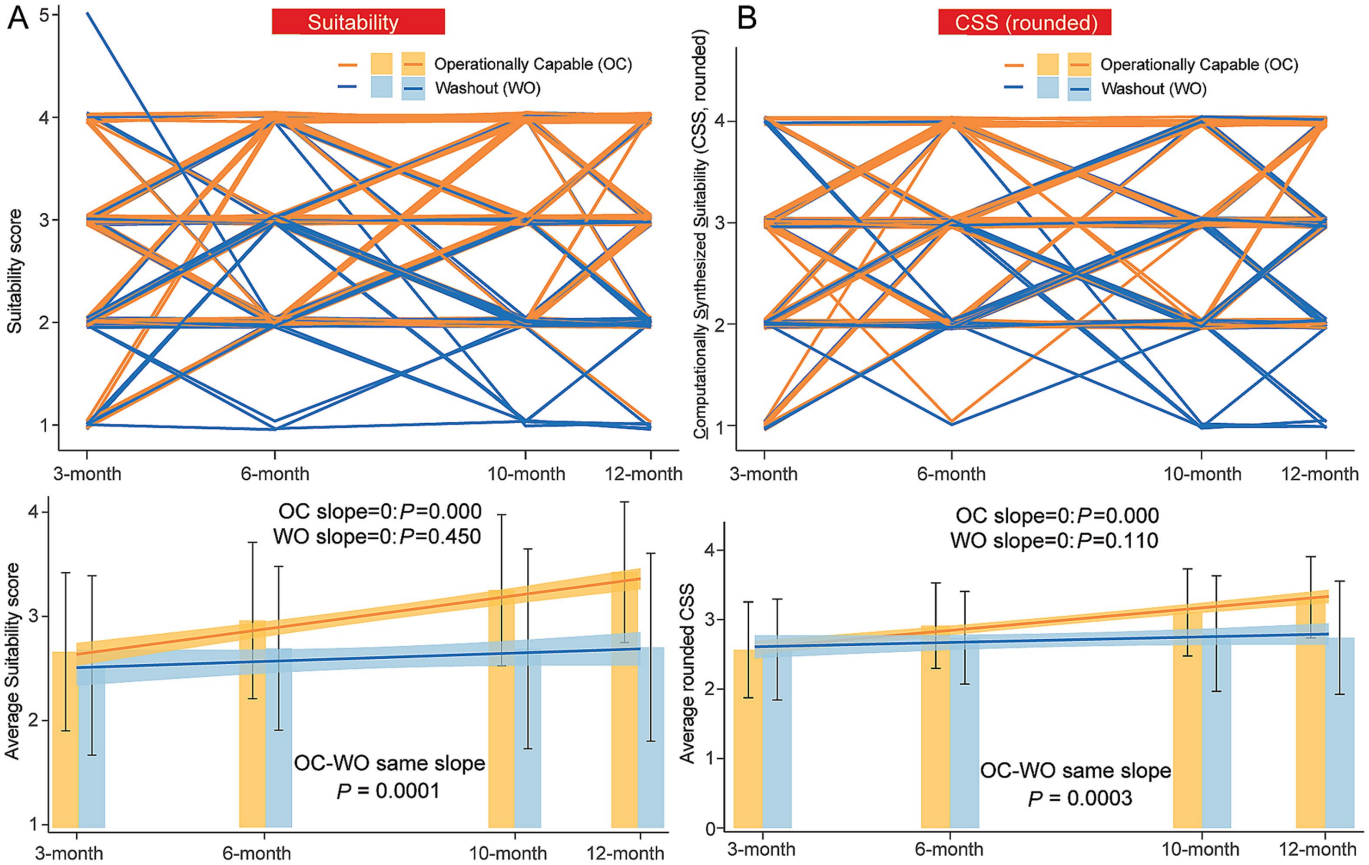
Changes in suitability scores across timepoints for operationally capable (OC) and washout (WO) dogs. Changes in evaluator-reported suitability scores **(A)** and computationally synthesized suitability (CSS) scores rounded to integer **(B)** across timepoints. *Top panel*, trajectory of suitability score changes for Operationally Capable (*orange*) and Washout dogs (*blue*). The *y*-axis is the ordinal behavioral evaluation scores ranging from 1 to 5. The *x*-axis represents the different timepoints (3-month, 6-month, 10-month, and 12-month). *Bottom panel*, barplots of average scores with error bars representing +/− SD at the 3-month, 6-month, 10-month, and 12-month timepoints for operationally capable (*orange*) and washout dogs (*blue*). A fitted line based on a linear regression model was plotted for each group (Operationally Capable: *orange*; Washout: *blue*) with a shared area representing 95% confidence interval. Statistical significance for whether the slope is zero is assessed by the Wilcoxon test. *p*-value for slope difference between operationally capable (OC) and washout (WO) groups is calculated using the Wilcoxon signed-rank test.

Because the predictive importance of suitability increased significantly from 3–6 months to 10–12 months (Figure 3; Table 1), we quantified the developmental changes in suitability using linear regression (see Materials and Methods). At the 3-month timepoint, no significant difference in suitability scores was observed between the OC and WO dogs (*p* > 0.05, Figure 5A). Interestingly, suitability scores remained unchanged in WO dogs (*p* = 0.45, Wilcoxon test), but improved significantly over time in OC dogs, resulting in an average 30% increase by 12 months (*p* = 0.0001; Figure 5A). CSS, which was predicted from five selected BCs using our established model (Figure 4), showed the same developmental pattern as suitability (Figure 5B).

**Figure 6:**
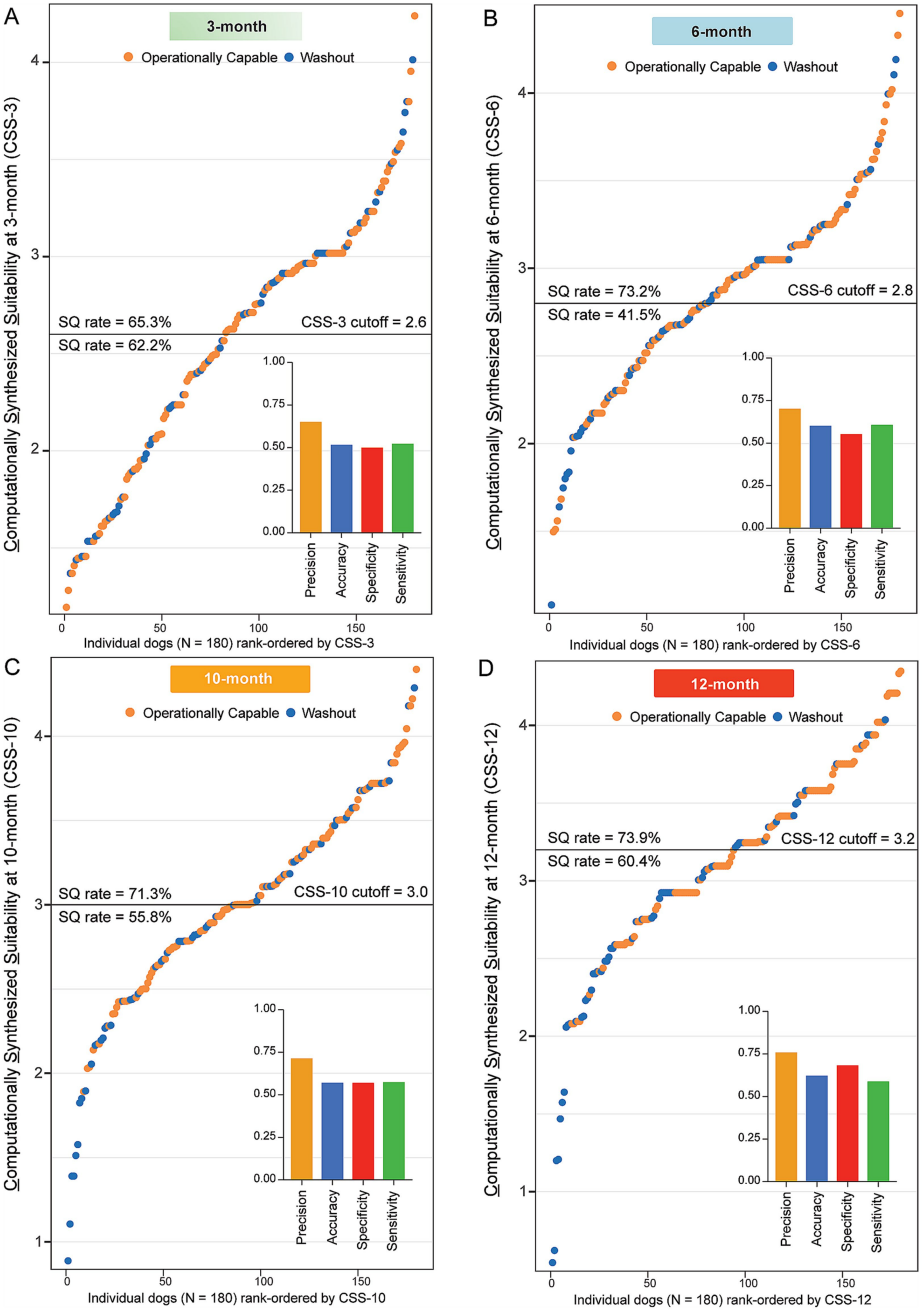
The performance of computationally synthesized suitability (CSS) scores as indicators for predicting detection dog training outcome. **(A–D)**. Rank-order plots illustrating the performance of computationally synthesized suitability (CSS) scores in predicting the training outcome of detection dogs at the 3-month **(A)**, 6-month **(B)**, 10-month **(C)**, and 12-month **(D)** timepoints. In each plot, individual dogs (*N* = 180, *x*-axis) are ranked from lowest CSS scores (*left*) to highest CSS scores (*right*). The *y*-axis shows the CSS score value ranging from 0 to 5. Each point is color-coded according to its training outcome (Operationally Capable: *orange*; Washout: *blue*). Barplots of key metrics evaluating the predictive model performance are included at the bottom right corner within each panel. Different cut-off lines are drawn in each panel (2.6 for CSS-3, 2.8 for CSS-6, 3.0 for CSS-10, and 3.2 for CSS-12).

Compared to the ordinal suitability score, CSS provided a standardized continuous measure of suitability (0–5), offering potential as a valuable tool for detection dog selection. We evaluated its utility for predicting detection dog training outcomes using empirically defined cutoffs at each developmental timepoint (Figure 6). CSS prediction performance improved with age, with accuracy increasing from 53% at 3 months (Figure 6A) to >75% precision from 10–12 months (Figures 6C,D). At 12 months, CSS achieved 76.7% precision, comparable to the model using all BCs combined (76.3%; Table 1). However, in terms of prediction accuracy, CSS-12 performed poorly (62%) compared to the evaluator-reported suitability (74%).

## Discussion

### Longitudinal behavioral phenotyping reveals the behavioral architecture underlying detection dog training success

Accurately and reliably evaluating a candidate detection dog’s suitability for operational work requires comprehensive behavioral assessments that capture the diverse superior characteristics of successful performance. Our dataset, including 24,133 standardized behavioral evaluations in 304 detection dogs, enabled a systematic characterization of the longitudinal relationships among suitability, BCs, and training outcome. The BCs were designed as standardized assessments with detailed scoring protocols to provide objective measures requiring minimal evaluator subjectivity. Their criterion validity has been demonstrated previously (7, 12), and the construct validity of several components has subsequently been confirmed in V2.0 (12, 23), although with some revision of scoring methodology as compared to V1.0 dataset used in this study.

Our longitudinal analyses further validated the behavioral assessment framework by demonstrating strong within-domain correlations among P-BCs and E-BCs, with relatively limited cross-domain connectivity. This distinct network structure supports the assignment of BCs into separate behavioral domains while revealing how these domains jointly contribute to operational suitability. The limited connectivity among E-BCs at the 3-month timepoint likely reflects greater behavioral variability in young puppies before environmental responses become more stable with maturation, consistent with the strengthening correlations observed at later developmental stages.

Several exceptions provide opportunities for further refinement of the assessment framework. Retrieve showed weaker correlation and was not connected to any other metric at the 6-month and 10-month timepoints. One possible explanation was that Retrieve has the least phenotypic variation among all behavioral components (Supplementary Figure S1), limiting the ability to detect meaningful relationships with other P-BC scores. Additionally, cross-domain connectivity between Work/Effort (P-BC) and Vehicles/Urban Clutter (E-BC) was observed at the 12-month timepoint, suggesting potential overlap between behavioral domains. Although these exceptions were uncommon, they highlight areas where additional efforts could further strengthen the construct validity of individual behavioral metrics.

### Evaluator-reported suitability integrates behavioral domains and is the strongest predictor of training success

Detection dog suitability refers to the extent to which a dog possesses the behavioral, cognitive, and physical traits required to successfully perform specific detection tasks in operational environments. It is inherently a composite phenotype spanning both the performance and environmental soundness domains contribute to operational success. Consistent with this concept, evaluator-reported suitability scores were the strongest behavioral predictor of training outcomes in this study, outperforming E-BC or P-BC scores alone at every timepoints, achieving ~74% accuracy at the 12-month timepoint.

The superior predictive performance of suitability likely reflects its ability to integrate information across multiple behavioral domains. Although environmental soundness and performance-related BCs formed largely independent correlation networks, suitability connected and bridged these two domains, particularly at later developmental stages. By the 12-month timepoint, suitability was the only behavioral metric significantly correlated with all BCs, effectively serving as a central hub of the behavioral network. In contrast, at the 3-month and 6-month timepoints, suitability was primarily associated with P-BCs, suggesting that environmental responses either had not yet stabilized or contributed less to evaluators’ overall assessments during early development.

Despite its strong predictive performance, evaluator-reported overall suitability remained a subjective assessment that was heavily relying on trainer experience and expertise. Potential sources of bias include inter-evaluator variability, trainers’ prior expectations, selective presentation of dogs to clients, and differences among clients’ operational requirements. However, our study was specifically designed to evaluate these potential confounders. We demonstrated that there was no significant variability among the three experienced trainers, and our training outcomes across dogs were determined by multiple independent clients, with OC dogs defined as those successfully sold to clients or, less frequently, by internal selection to retain for breeding. Furthermore, evaluators assigned overall suitability scores after completing standardized behavioral component assessments, suggesting that suitability represents an integrated summary of multiple objectively measured behavioral traits rather than an entirely subjective impression.

Taken together, these findings indicate that evaluator-reported suitability provides a robust, integrated measure of operational potential by synthesizing information across multiple behavioral domains. This observation motivated the development of CSS, an objective statistical framework designed to capture the same behavioral information using standardized BC scores.

### Computationally synthesized suitability decomposes and recapitulates evaluator-reported suitability

Although evaluator-reported detection dog suitability effectively predicted detection dog training outcomes, the relative contribution of individual BCs remained unclear. To quantify these contributions, we developed Computationally Synthesized Suitability (CSS), a linear model that estimates evaluator-reported suitability from BC scores and enables the relative importance of individual behavioral traits to be quantified across developmental stages. This approach allowed us to quantify the weight assigned to specific traits and examine how these weights shift across developmental timepoints.

CSS modeling demonstrated that the evaluator-reported suitability was not dominated by any single BC but instead integrates information from five significant BCs. At 3 months, P-BC factors, including Work/Effort, Hunt, Toy. P, and Independent, contributed most strongly to CSS, whereas environmental soundness-related BCs became increasingly important from 6 months onward, consistent with the strengthening correlations between suitability and environmental soundness observed during development. These findings indicated that trainers progressively incorporated environmental soundness into their assessments as the dogs matured.

CSS closely recapitulated evaluator-reported suitability across all developmental timepoints (Spearman’s *ρ* = 0.84–0.89). The slightly stronger agreement at earlier stages suggests that evaluator judgments relied primarily on standardized behavioral assessments when limited individual experience with each dog was available. As dogs matured, evaluators likely incorporated additional qualitative observations acquired through daily interactions, explaining the modest reduction in CSS-suitability correlations at later developmental stages. Together, these findings demonstrate that CSS provides an objective statistical approximation of evaluator-reported suitability while revealing how trainers integrate behavioral information during detection dog development.

### Developmental trajectories of suitability distinguish successful from unsuccessful detection dogs

We observed a progressive increase in the accuracy of predicting training outcomes from behavioral metrics across timepoints, consistent with previous literature demonstrating that the selection of successful candidate working dogs improves as dogs mature (12, 24–26). Our data also revealed that suitability increases over time in OC dogs, but not in WO dogs. Notably, OC and WO dogs showed equivalent suitability scores at the earliest 3-month timepoint, with significant differences only occurring at the 10-month timepoint and later. This divergence between successful and unsuccessful dogs was also previously observed in this population (7) and other detection dogs (14). In this study, we have shown that in OC dogs, the observed improvement in suitability across developmental timepoints may be driven, in part, by the progressive enhancement of individual BCs, particularly E-BC related phenotypes. The developmental plasticity of E-BC phenotypes we observed may be an important factor distinguishing OC and WO dogs.

### Other biological and environmental factors influencing training success

While certain behavioral phenotypes were shown to predict the training success of detection dog, other factors may also play significant roles. At the 3-month and 6-month timepoints, litter identity is the most important factor in predicting training outcomes. In other words, the best predictor for any dog’s success is the success rate of its own littermates, suggesting that the genetic predisposition is also crucial for detection dogs’ success. These factors were particularly important for the earliest timepoints when scores from the behavioral components were still developing and not as informative. In Australian Labrador retrievers, chocolate coat color was shown to be associated lower trainability (27). However, we observed the opposite in our dataset: chocolate coat color was associated with a higher success rate, but this association was driven by a small subset of closely related OC chocolate dogs. Therefore, this effect likely reflects the influence of litter identity rather than a true effect of coat color. Additionally, sex, delivery type, and the identity of the evaluator did not exhibit significant predictive power for training outcomes.

### Applications, limitations, and future directions

Our results demonstrated that evaluator-reported suitability provided a practical metric for predicting detection dog training outcomes by integrating behavioral information across both performance and environmental soundness domains. However, its predictive value depended on the developmental stage. The distinct correlation structures observed at the 3- and 6-month timepoints, compared with the highly connected networks at the 10- and 12-month timepoints, suggested that behavioral responses were less stable during early development. Consequently, suitability assessments performed at 10-month or later timepoint provided substantially greater predictive accuracy and were likely to be the most useful for operational decision-making.

Further extending this concept, CSS-12 provided an objective estimate of evaluator-reported suitability using five standardized BC phenotypes. Because CSS was derived from quantitative behavioral assessments, it reduced subjectivity and potential inter-evaluator variability while providing a continuous suitability measure, enabling finer discrimination among dogs. CSS-12 achieved a precision of 76.7%, which was comparable to models incorporating all behavioral components, demonstrating its potential as a standardized decision-support tool for detection dog selection.

Despite these advantages, CSS also had important limitations. Restricting the model to five behavioral components may overlook additional factors contributing to detection dog success, and although CSS demonstrated high precision, its overall prediction accuracy remained lower than that of evaluator-reported suitability. Furthermore, both suitability and CSS should be interpreted within the context in which they were developed. Evaluator-reported suitability relies on experienced trainers who understand the behavioral requirements of specific detection tasks, whereas the performance of CSS should be validated across independent populations, training programs, and operational settings before broader implementation.

Detection dog training success is influenced by a combination of multiple factors beyond behavioral assessments alone. While suitability is a key predictor of training success, it is not the sole determinant. Other factors, such as cognitive traits (28–31), physical soundness (15), nutrition (32), gut microbiota (33–35), and nasal microbiota (36), also contribute to behavior and cognition, influencing detection dog success. Future predictive models should integrate these factors with the behavioral insights identified in this study. For example, developmental improvements in suitability, which distinguished OC dogs from washouts, could be incorporated into longitudinal prediction models, while refined BC panels may further improve CSS. Ultimately, integrating longitudinal behavioral phenotypes with genomic information will enable the development of comprehensive phenomics-genomics models that provide a more complete understanding of the biological basis of detection dog performance and facilitate more accurate selection and breeding strategies.

## Conclusion

Our study provides a comprehensive longitudinal characterization of the behavioral architecture underlying detection dog training success. By integrating more than 24,000 standardized behavioral evaluations from 304 detection dogs, we demonstrate that evaluator-reported suitability is the strongest behavioral predictor of operational outcomes because it synthesizes information across both performance-related and environmental soundness-related behavioral domains. We further show that Computationally Synthesized Suitability (CSS) closely recapitulates evaluator-reported suitability while providing a standardized, objective framework for quantifying operational suitability from individual BCs. Rather than simply predicting detection dog success, CSS objectively and reproducibly approximates the holistic assessments made by experienced trainers, thereby transforming complex behavioral phenotypes into a quantitative measure of operational suitability. By integrating multiple behavioral components across developmental stages, this framework captures functional manifestations of the cognitive and behavioral processes that underlie working dog performance. These findings highlight the importance of longitudinal behavioral assessment and demonstrate that predictive accuracy improves substantially as dogs mature. Collectively, our results establish an evidence-based framework for objectively evaluating detection dog suitability while advancing quantitative behavioral assessment methodologies. More broadly, this work provides a standardized, reproducible approach for studying complex behavioral phenotypes and animal cognition, reducing reliance on subjective evaluations and facilitating more rigorous comparisons across studies. Future integration of longitudinal behavioral phenotypes with genomic data and other biological measurements will enable comprehensive phenomics-genomics approaches to further improve the selection, breeding, and training of detection dogs.

## Data Availability

The original contributions presented in the study are included in the article/Supplementary material, further inquiries can be directed to the corresponding author. All codes used in this study are available at https://github.com/Ginny-Zheng/Linking-trainer-reported-assessments-of-detection-dog-suitability-with-standardized-behavioral-tests.

## Supplementary material

SUPPLEMENTARY TABLE S1 Selected behavioral component phenotypes for estimating suitability scores at 3-month, 6-month, 10-month, and 12-month timepoints.

SUPPLEMENTARY FIGURE S1 Distribution of behavioral metrics and other phenotypic features in detection dogs.

SUPPLEMENTARY FIGURE S2 Random forest models for detection dog training outcome prediction revealed no significant evaluator bias.

SUPPLEMENTARY FIGURE S3 Inter-developmental timepoints correlative structures of predicted suitability (CSS) scores and 11 behavioral component phenotype scores.

SUPPLEMENTARY FIGURE S4 Similarities in the correlative structures of evaluator-reported suitability scores and computationally synthesized suitability (CSS) scores with the 11 behavioral component phenotypes at the 3-month, 6-month, and 10-month timepoints.
