# Supplementary material for: Behavioral components define operational suitability metric for detection dog success
Source: Front Vet Sci. 2026 Jul 27;13:1887925. doi: 10.3389/fvets.2026.1887925 (PMC13454109; doi:10.3389/fvets.2026.1887925)
Supplement: Supplementary file 1 [file Image_1.pdf]

Figure S1. Distribution of behavioral metrics and other phenotypic features in detection dogs.

(**A**) Stacked barplot of the number of dogs born in each year between 2012 and 2018 binned by training outcome (operationally capable vs. washout). (**B**) Stacked barplot of sex composition of detection dog cohort binned by training outcome (operationally capable vs. washout). (**C**) Stacked barplot of the number of dogs born through cesarean sections and free whelping binned by training outcome (operationally capable vs. washout). (**D**) Stacked barplot of the number of dogs with different coat colors (black, chocolate, and yellow) binned by training outcome (operationally capable vs. washout). (**E**) Barplots showing the distributions of evaluation scores for 12 behavioral metrics. Each bar is color-coded by the training outcome (operationally capable vs. washout). Three categories of metrics are included: performance-related (P) metrics, environmental soundness-related (E) metrics, and suitability scores. P-BC scores include Air scenting, Work/Effort, Independence, Physical Possessiveness of Toy, Task/RewardFocus on Task/Rewards, Retrieve, and Hunt. E-BC scores include Surfaces, People, Vehicles/Urban Clutter, and Excitability.

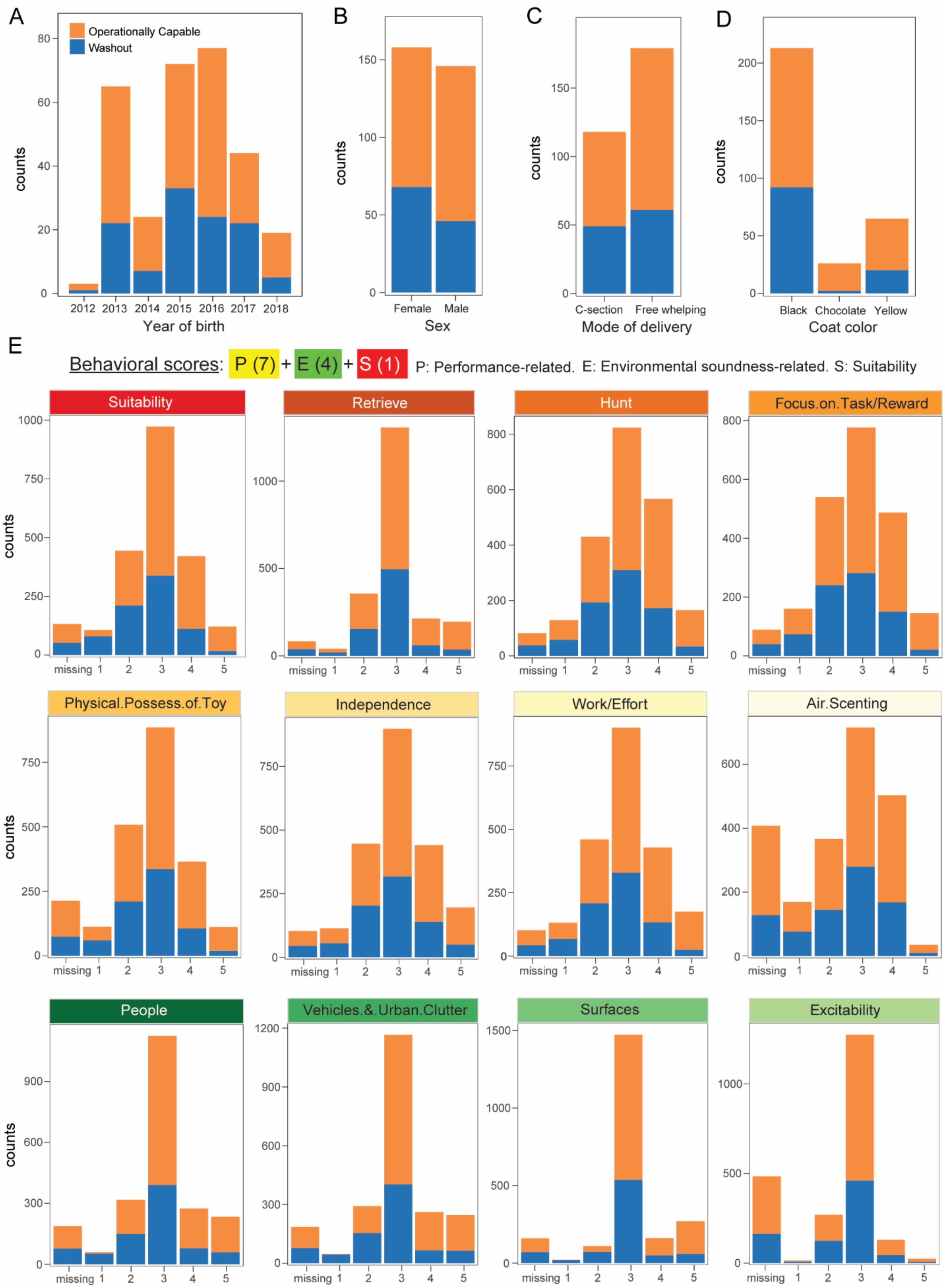
A
B
C
D
E
Operationally Capable
Washout
counts
Year of birth
Sex
Mode of delivery
Coat color
Female
Male
C-section
Free whelping
Black
Chocolate
Yellow
Behavioral scores: P (7) + E (4) + S (1)
P: Performance-related. E: Environmental soundness-related. S: Suitability
Suitability
Retrieve
Hunt
Focus.on.Task/Reward
Physical.Possess.of.Toy
Independence
Work/Effort
Air.Scenting
People
Vehicles.&.Urban.Clutter
Surfaces
Excitability
missing
