# Supplementary material for: Behavioral components define operational suitability metric for detection dog success
Source: Front Vet Sci. 2026 Jul 27;13:1887925. doi: 10.3389/fvets.2026.1887925 (PMC13454109; doi:10.3389/fvets.2026.1887925)

Figure S2. Random forest models for detection dog training outcome prediction revealed no significant evaluator bias.
Barplots of feature importance scores computed using random forest regression at the 3-month, 6-month, 10-month, and 12-month timepoints using all variables and all datapoints from 304 dogs. Longitudinal phenotypic measurements included in the model are 7 performance-related behavioral component phenotypes (P-BC), 4 environmental soundness-related behavioral component phenotypes (E-BC), and suitability scores. Invariant phenotypes and features included are dam and sire's age at birth, litter ID, coat color, sex, delivery type, and evaluator. Lines shaded in gray connect the Evaluator variable to demonstrate its change in feature importance ranking across developmental timepoints.

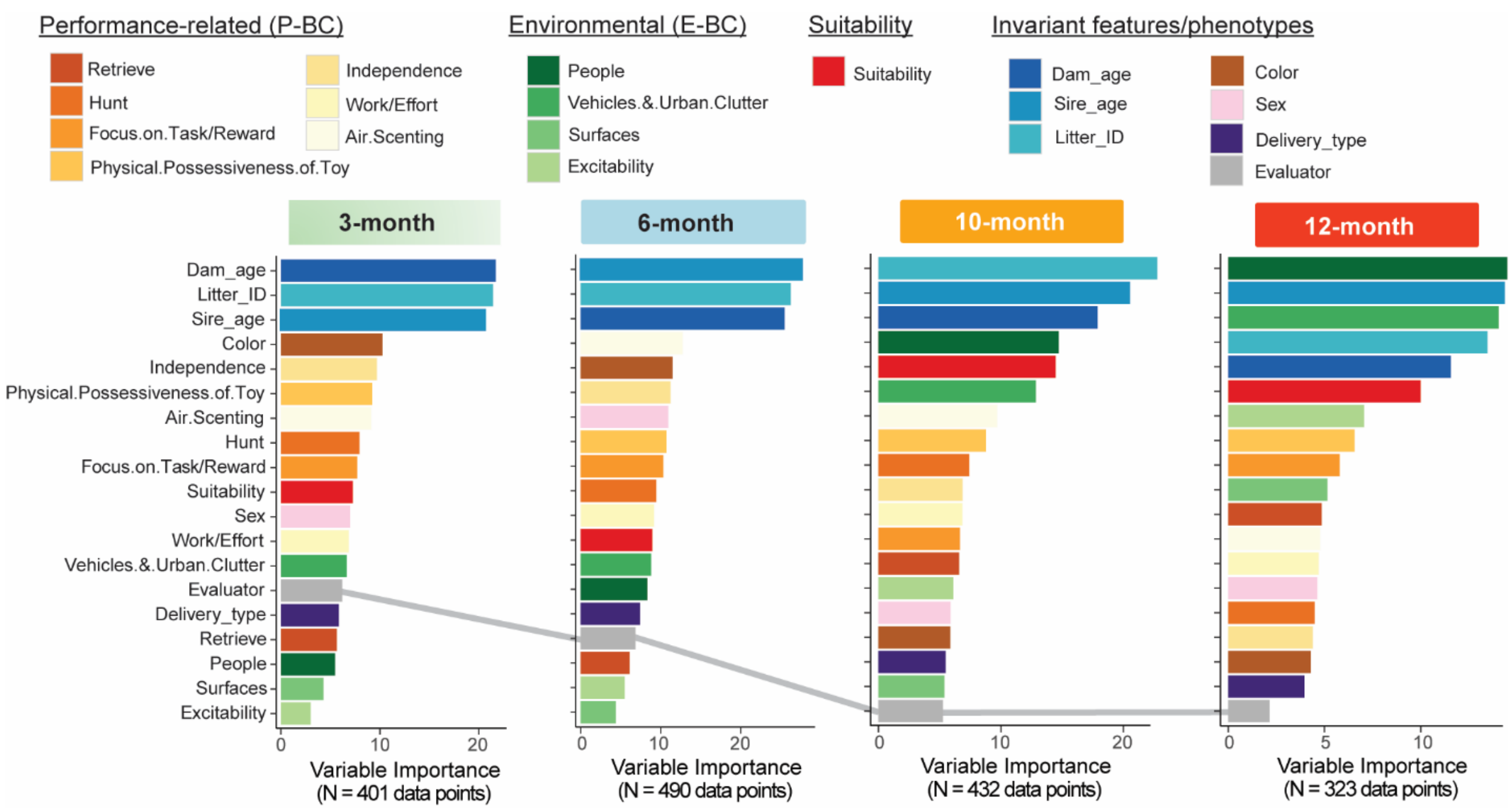

Supplement: Supplementary file 2 [file Image_2.pdf]
