# Supplementary material for: Behavioral components define operational suitability metric for detection dog success
Source: Front Vet Sci. 2026 Jul 27;13:1887925. doi: 10.3389/fvets.2026.1887925 (PMC13454109; doi:10.3389/fvets.2026.1887925)

Figure S3. Inter-developmental timepoints correlative structures of predicted suitability (CSS) scores and 11 behavioral component phenotype scores.

Correlation network plots demonstrating significant correlations among CSS (computationally synthesized suitability) scores and 11 behavioral component (BC) phenotype scores at the 3-month, 6-month, 10-month, and 12-month timepoints. Performance-related BC phenotypes (P-BC), including Air.Scenting, Work/Effort, Independence, Physical Possessiveness of Toy (Toy.P), Focus on Task/Reward (Task.F), Retrieve, and Hunt, are represented by circles color-coded using a yellow-brown spectrum, with age in months shown in the middle. Environmental soundness-related BC phenotypes (E-BC), including Surfaces, People, Vehicles/Urban Clutter, and Excitability, are represented in green circles. Red circles represent CSS scores, which are moved inside the circle for better visualization. The paternal age and maternal age at birth are represented by blue circles. Statistically significant Spearman's correlations exceeding a predefined threshold of 0.5 are depicted by edges connecting the respective variables. The color of the edge indicates the sign of the correlation, with red representing positive correlations and blue indicating negative correlations. The five BC phenotypes used in estimating CSS scores are highlighted by black boxes.

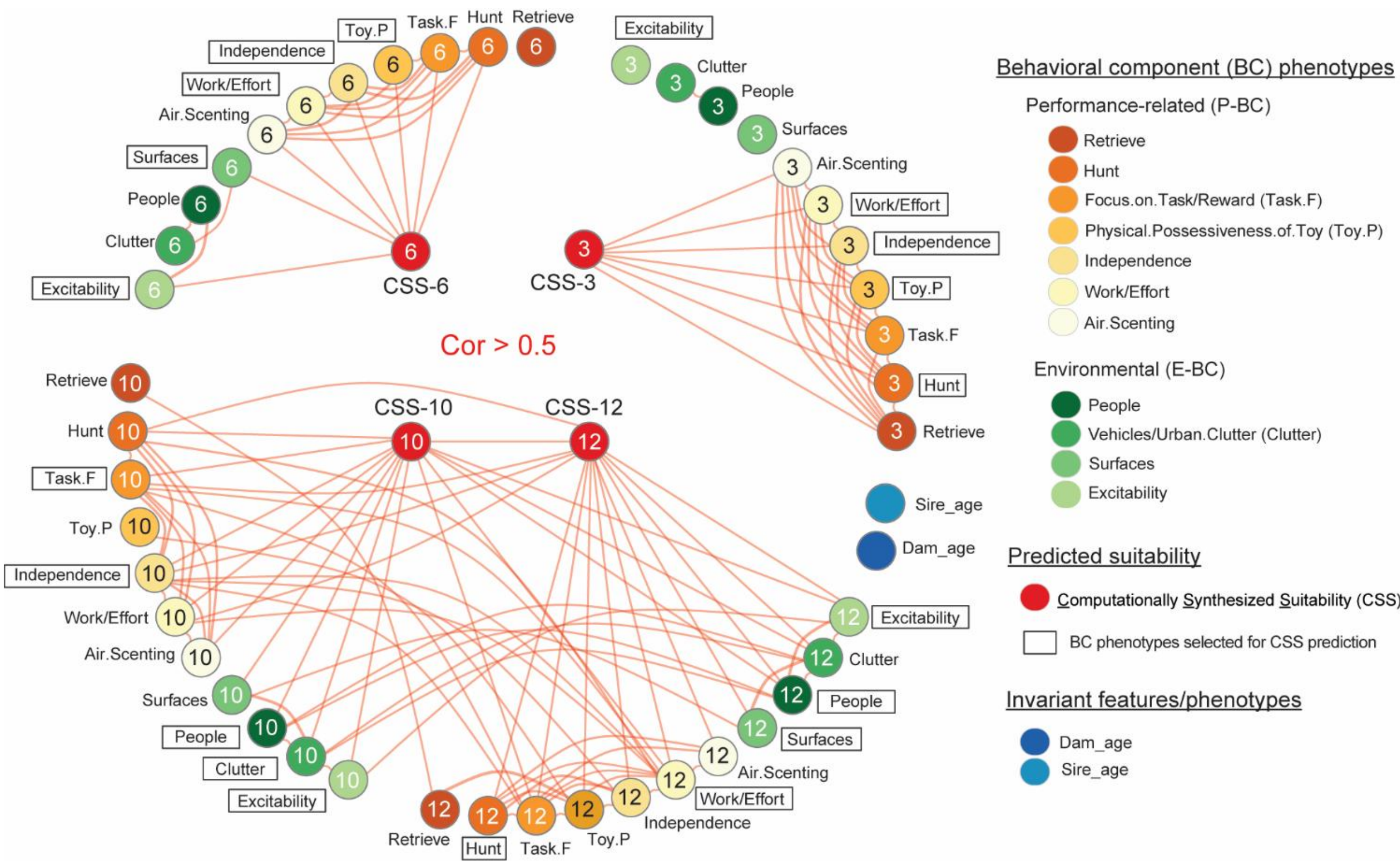

Supplement: Supplementary file 3 [file Image_3.pdf]
