# Supplementary material for: Behavioral components define operational suitability metric for detection dog success
Source: Front Vet Sci. 2026 Jul 27;13:1887925. doi: 10.3389/fvets.2026.1887925 (PMC13454109; doi:10.3389/fvets.2026.1887925)

Figure S4. Similarities in the correlative structures of evaluator-reported suitability scores and computationally synthesized suitability (CSS) scores with the 11 behavioral component phenotypes at the 3-month, 6-month, and 10-month timepoints.

Correlation network plots demonstrating significant correlations among evaluator-reported suitability scores (**A**) or CCS scores (**B**) and 11 behavioral component (BC) phenotypes at the 3-month, 6-month, and 10-month timepoints. Performance-related behavioral component phenotypes (P-BC), including Air.Scenting, Work/Effort, Independence, Physical Possessiveness of Toy, Focus on Task/Reward, Retrieve, and Hunt, are represented by circles color-coded using a yellow-brown spectrum. Environmental soundness-related behavioral component phenotypes (E-BC), including Surfaces, People, Vehicles/Urban Clutter, and Excitability, are represented in green circles. Red circles represent evaluator-reported suitability scores (**A**) or CSS scores (**B**). The paternal age and maternal age at birth are represented by blue circles. Statistically significant Spearman's correlations exceeding a predefined threshold of 0.5 are depicted by edges connecting the respective variables. The color of the edge indicates the sign of the correlation, with red representing positive correlations and blue indicating negative correlations.

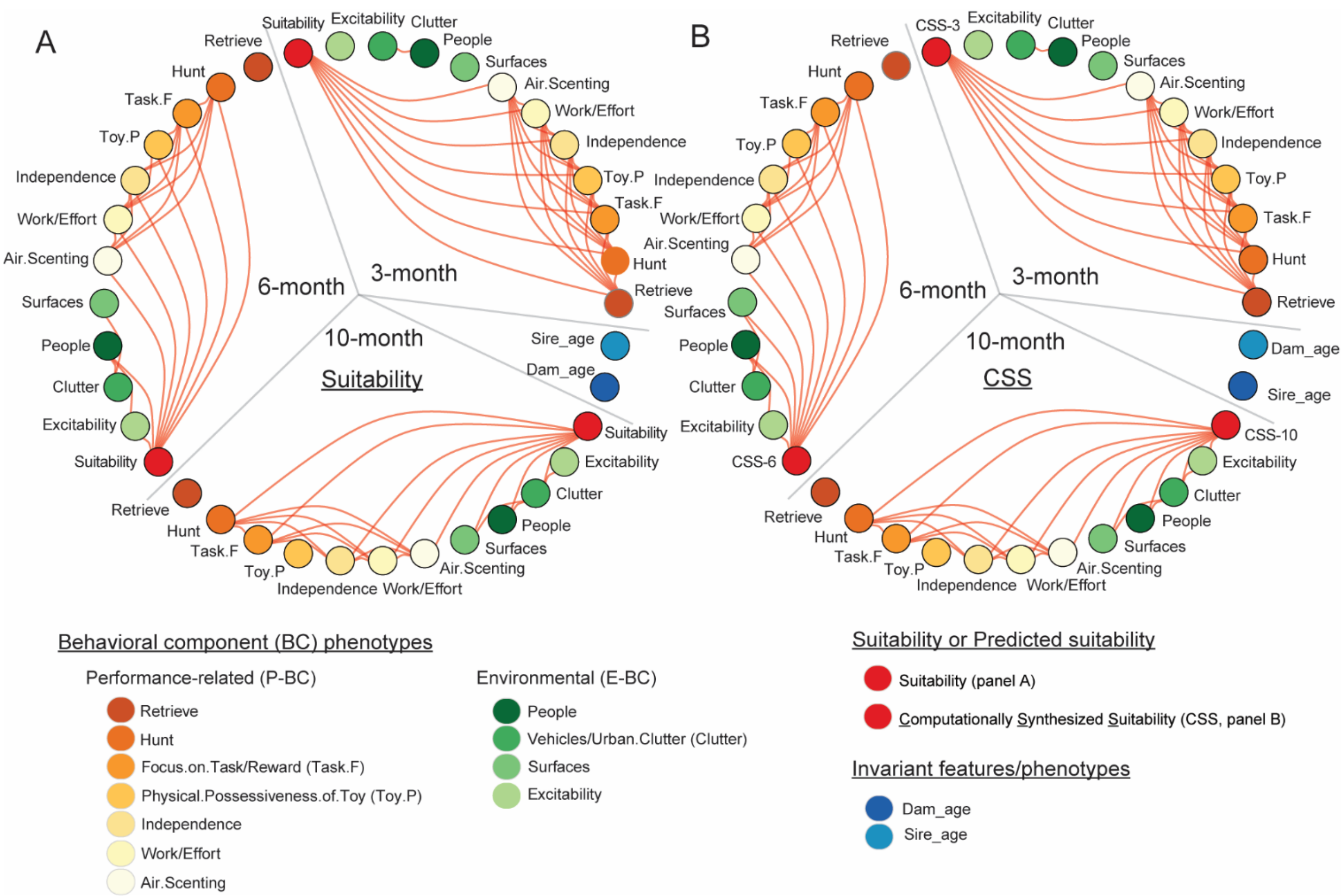

Supplement: Supplementary file 4 [file Image_4.pdf]
