# Supplementary material for: Behavioral components define operational suitability metric for detection dog success
Source: Front Vet Sci. 2026 Jul 27;13:1887925. doi: 10.3389/fvets.2026.1887925 (PMC13454109; doi:10.3389/fvets.2026.1887925)
Supplement: Supplementary file 5 [file Table_1.pdf]

Supplemental Table S1. Selected behavioral component phenotypes for estimating suitability scores at 3-month, 6-month, 10-month, and 12-month timepoints.

3-month	*P*-value	6-month	*P*-value	10-month	*P*-value	12-month	*P*-value
Work/Effort	<0.00001	Work/Effort	<0.00001	Task.F*	<0.00001	Work/Effort	<0.00001
Hunt	0.00012	Surfaces	<0.00001	Independence	0.00001	Hunt	<0.00001
Toy.P*	0.00026	Toy.P*	0.00012	People	0.00005	Surfaces	0.00018
Excitability	0.02528	Excitability	0.00019	Clutter*	0.00036	People	0.00294
Independence	0.03878	Independence	0.00102	Excitability	0.00114	Excitability	0.01567

* **Toy.P**: Physical Possessiveness of Toy. **Task.F**: Focus on Task/Reward. **Clutter**: Vehicles/Urban Clutter.
